# Chief Financial Officer Functional Diversity and the Timeliness of Annual Reports: A Comparative Study of Firms With Different Ownership Types

**DOI:** 10.3389/fpsyg.2022.889007

**Published:** 2022-05-09

**Authors:** Hao Yu, Weiguang Huang

**Affiliations:** School of Management, Zhejiang University of Technology, Hangzhou, China

**Keywords:** CFO, functional diversity, China, ownership type, timeliness of annual reports

## Abstract

Functional diversity is related to functional areas in which CFOs are experienced. It reflects their number of general managerial skills or social ties to some extent. In this paper, we try to examine whether there is an association between CFO functional diversity and the timeliness of annual reports. Using data on Chinese listed firms from 2009 to 2017, we found that in state-owned enterprises, there is a negative relationship between this diversity and timeliness. However, the promotion incentive of CFOs with functionally diverse experience can weaken this negative relationship. We also found that there is a positive relationship between the two factors in private enterprises whose offices are headquartered in regions with a low degree of marketization. We use difference-in-differences method to test the hypotheses again. The conclusions remain robust.

## Introduction

Since 2007, the China Securities Regulatory Commission (CSRC) has set a time limit for the disclosure of annual reports. It requires listed firms to make their annual reports public before the end of April of the following year (Cao and Wang, 2021). Chief financial officers (CFOs) are responsible for the preparation of financial statements, which are an essential part of annual reports. Do CFOs affect the disclosure of annual reports? If so, would the timing of disclosure be different for CFOs with different characteristics? And if it is, would the association between the two factors vary under different institutional backgrounds?

We focus on CFOs’ functional experience. Most CFOs start their careers in an accounting or cashier position. They may then have the opportunity to work in different functional areas, such as management, marketing, and production. The more diverse their functional experience, the wider their social connections and the more comprehensive their general managerial skills. Thus, they are likelier to exhibit heterogeneity in decision-making (Buchheit et al., 2019).

In China, state-owned enterprises (SOEs) are quite different from private enterprises (PEs) (Milhaupt, 2020). SOEs are part of the Chinese planned economy, and they retain the imprint of socialism. They must take on many social responsibilities. In addition, they are subject to supervision and control by the government (Kong et al., 2020). In contrast, PEs pursue fewer goals and are more independent.

We investigate the relationship between CFOs’ functional diversity and the timeliness of annual reports separately for SOEs or PEs. The empirical results showed that CFOs’ functional diversity is associated with the timeliness. Moreover, under different institutional backgrounds, the relationship varies greatly. We also observe that, in SOEs, the personal motivation of CFOs with functionally diverse experience moderates the degree of relationship. In PEs, the external environment affects the significance of linkage.

The remainder of the paper is organized as follows. We first review the literature on generalist executives and financial reporting timeliness. On this basis, we develop the research hypotheses. Subsequently, we detail the research design and present the statistical results. The last part discusses the findings and the limitations of the study.

## Literature Review

### Literature on Generalist Executives

The more functional areas the executive has experience in, the more general managerial skills he or she may learn. Accordingly, he or she can be regarded as a generalist. Research on generalist executives usually revolves around CEOs. Due to complementary human capital, generalist CEOs can play a distinctive role. For example, they are more willing to conduct research and development activities (Custódio et al., 2019); more inclined to conduct resource-integration business, such as mergers or acquisitions (Xu et al., 2021); and more positively associated with strategic distinctiveness (Crossland et al., 2014).

On the other side of the coin, they must take more risks. Consequently, their firms may have a lower credit rating (Ma et al., 2021a), higher equity costs (Mishra, 2014), or higher audit fees (Ma et al., 2021b). Some scholars have focused on the relationship between generalist CEOs and their compensation. They found that generalist CEOs have been paid a premium (Custódio et al., 2013). Other scholars have also found that when the complementary advantage of generalist CEOs is not evident, the premium will also disappear (Mueller et al., 2021).

Some scholars have focused on the effect of executive functional diversity on teamwork. They found that the more diverse the executive functional experience, the more information shared across the team, and the higher their involvement in decision-making (Bunderson and Sutcliffe, 2002; Bunderson, 2003). In an environment with high uncertainty, the participation of generalist executives can significantly improve firm performance (Cannella et al., 2008).

### Literature on Financial Reporting Timeliness

Different scholars have studied the timeliness of annual reports from different perspectives. Using data from Chinese listed firms, Haw et al. (2000) found that good news firms release their annual reports earlier than bad news firms, and loss firms release their annual reports the latest. A recent study by Bannouh et al. (2019) showed similar results. Li et al. (2020) found that firms strategically change disclosure timing to hide bad news (for example, from January to April), and this opportunistic behavior may increase future stock price crash risk. Selleslagh et al. (2021) found a negative association between firms’ financial health and late filing. However, their research also showed a positive relationship for firms of late filing consistently.

It is worth noting that few scholars investigate the association between human capital attributes and the timeliness of financial reporting. Abernathy et al. (2018) is an exception. They found that the higher managerial ability is associated with a shorter earnings announcement lag, a shorter audit report lag, and a lower probability of late filing.

## Hypothesis Development

SOEs seem like small, closed societies. According to Lin (2013) statistics, among the top 100 Chinese state-owned listed firms, 82% of executives’ career trajectories came from only one firm or business group. This implies that executives at SOEs always develop their careers from within the firm or business group. For CFOs, the more diverse their functional experience, the wider the internal social networks they may develop. As a result, they may be less worried about their occupations.

However, the broad internal networks also reduce the cost of information transmission. Consequently, it becomes easier for upper-level leaders (e.g., CEOs) to intervene in CFOs’ financial reporting work. The more their work is interfered with, the less motivated they will be to make decisions, which would lead to counterproductive activities (Fan et al., 2007), provided that their interests are not damaged. That is, CFOs with more internal social resources may be less proactive in preparing financial statements.

The manager-owner agency problem is serious in Chinese SOEs (Fan et al., 2007; Fang et al., 2018). Under this institutional context and combined with the above analysis, we propose the first hypothesis:

**Hypothesis 1:** Other things being equal, the more diverse the functional experience of CFOs at SOEs, the less timely the disclosure of annual reports.

In SOEs, promotion is the managers’ main incentive (Jiang and Kim, 2020). While functionally diverse experience can provide generalist CFOs an advantage for promotion, it is not enough. Other requirements are to meet, such as academic degree, age, and/or work experience (Leutert and Vortherms, 2021).

Late filing is possibly linked with a series of adverse outcomes, such as financial restatements or stock price crashes (Ma et al., 2018; Li et al., 2020), which may potentially destroy the bright future of those generalist CFOs who have promotion incentive. To reduce such threats, they may speed up their paperwork. Based on Hypothesis 1, we then propose the second hypothesis:

**Hypothesis 2:** Other things being equal, the promotion incentive of functionally diverse experienced CFOs at SOEs will weaken the negative association between functional diversity and the timeliness of annual reports.

The concept of efficiency is deeply rooted in PEs (Howell, 2020). Under the principle of efficiency first, functionally diverse experience does not guarantee CFOs’ career security or chance of promotion. On the contrary, their capability will be questioned if they cannot demonstrate their complementary value as human capital. So, to maintain professional legitimacy or personal reputation, generalist CFOs at PEs may be more proactive in preparing financial statements.

Market development in the region can undermine generalist CFOs’ complementary value in financial reporting. On the one hand, the higher the level of marketization in the region, the more complex the firm’s businesses will become. Consequently, generalist CFOs will spend less time preparing financial statements for additional non-accounting tasks (e.g., financing, mergers, and acquisitions).

On the other hand, the higher the level of marketization in the region, the more likely generalist CFOs’ work can be replaced by other resources. Thus, the complementary value of generalist CFOs decreases due to resource substitutability.

In short, there may be a positive association between functional diversity and timeliness for PEs in less market-developed regions. We then propose the third hypothesis:

**Hypothesis 3:** Other things being equal, in less market-developed regions, the more diverse the functional experience of CFOs at PEs, the timelier the annual report.

## Research Design

### Models and Variables


(1) $${\text{DELAY}}_{\text{i},\text{t}}=\beta_{0}+\beta_{1}\,{\text{DIV}}_{\text{i}},\text{t}+\Sigma \,\rho_{\text{i}}\,{\text{CONTROL}}_{\text{i}},\text{t}+\tau_{\text{i}}+\gamma_{\text{t}}+\varepsilon_{\text{i},\text{t}}$$

(2) $${\text{DELAY}}_{\text{i},\text{t}}=\beta_{0}+\beta_{1}\,{\text{DIV}}_{\text{i},\text{t}}+\beta_{2}\,{\text{INCEN}}_{\text{i},\text{t}}+\beta_{3}\,{\text{INCEN}}_{\text{i},\text{t}}*{\text{DIV}}_{\text{i},\text{t}}+\Sigma \,\rho_{\text{i}}\,{\text{CONTROL}}_{\text{i},\text{t}}+\tau_{\text{i}}+\gamma_{\text{t}}+\mu_{\text{i},\text{t}}$$

To test the above hypothesis, we built two models. Model (1) is used to test Hypotheses 1 and Hypotheses 3, while Model (2) is used to test Hypothesis 2.

Regarding timeliness, we use the number of days between the fiscal year-end and the actual disclosure date as the first indicator (DELAY1): the smaller this indicator, the timelier the annual reports.

At the end of each fiscal year, Chinese listed firms must inform the Shanghai or Shenzhen Stock Exchange of the scheduled disclosure date of annual reports. Many firms (18.74% in our sample) do not disclose annual reports as planned for various reasons, such as avoiding misreporting or waiting for (or following) their within-industry peers (Ma et al., 2018; Cao and Wang, 2021). To remove the possible noise that this time interval may bring, we use the number of days between the fiscal year-end and the originally scheduled disclosure date as the second indicator of timeliness (DELAY2).

Functional diversity is the number of functional areas CFOs experience minus 1. Ten functional areas are identified in the China Stock Market and Accounting Research (CASMAR) database: (1) Production, (2) research, (3) design, (4) human resources, (5) management, (6) marketing, (7) finance, (8) accounting, (9) law, and (10) other or unclear. Because category (10) is difficult to understand, it is not included in our analysis.

Regarding promotion incentive, we use two indicators. If generalist CFOs are younger and have a bachelor’s degree or higher (INCEN1), they may have a better chance of promotion. Another indicator is concerned with social capital. If generalist CFOs have social ties with governments or related institutions (INCEN2), their chances of promotion may also be enhanced. This is because the governments or related institutions are the ultimate controllers of personnel arrangements in SOEs.

We choose several types of control variables. First, other demographic characteristics of CFOs may affect timeliness, such as tenure (TEN), age (AGE), whether they are also firm’s directors (DIR) and have certified public accountant licenses (CPA) (Bedard et al., 2014; Ghafran and Yasmin, 2018). Second, we consider the firm’s basic characteristics (SIZE, LEVER, ROA) and business complexity (INTAN, INVEN, REC) (Abernathy et al., 2018; Ma et al., 2018). Timeliness may be negatively affected if a firm’s profitability declines or loss occurs. We thus add BADNEWS and LOSS (Sengupta, 2004; Abernathy et al., 2018; Cao and Wang, 2021). The larger the subsidiaries, the slower the information will be collected. Thus, we add SUBS (Ma et al., 2018). The proprietary costs (MKBK) may also be associated with timeliness (Sengupta, 2004).

In addition to the above variables, we also consider variables related to the governance environment. Among them, the variables associated with the internal governance environment include LARGEST, BALANCE, INDEP, and BOARD (Ghafran and Yasmin, 2018; Cao and Wang, 2021); the variables related to the external governance environment include NAF, AO, BIG4, and REGION (Ma et al., 2018; Chen et al., 2020). The definitions of these variables are shown in Table 1.

**TABLE 1:** Variable definition.

Variable	Definition	Data source
DELAY1	Days between the fiscal year-end and the actual annual report disclosure date	CASMAR database
DELAY2	Days between the fiscal year-end and the originally scheduled annual report disclosure date	CASMAR database
DIV	Number of CFO’s functional backgrounds minus one.	CASMAR database
INCEN1	If the CFO’s age is less than the average age of executives and directors and the CFO has a bachelor’s degree or higher, 1; others, 0	CASMAR database
INCEN2	If CFO has social ties in governments or related institutions, 1; or, 0	CASMAR database
TEN	Tenure (Years) of CFO	Manual collection
AGE	Age (Years) of CFO	CASMAR database
DIR	If CFO is also a director of the board, 1; or 0	CASMAR database
CPA	If CFO is also a Certified Public Accountant, 1; or 0	CASMAR database
SIZE	Natural logarithm of assets	CASMAR database
LEVER	Liabilities/assets	CASMAR database
ROA	Net income/average assets	CASMAR database
INTAN	Intangible assets divided by total assets	CASMAR database
INVEN	Inventory divided by total assets	CASMAR database
REC	Accounts receivable divided by total assets	CASMAR database
BADNEWS	If ROA at year T is less than ROA at T-1, 1; or 0	CASMAR database
LOSS	If net income is negative, 1; or 0	CASMAR database
SUBS	The natural logarithm of the number of subsidies	Manual collection
MKBK	The ratio of market value of equity to book value of equity	Manual collection
LARGEST	The shareholding ratio of the first-largest shareholder	CASMAR database
BALANCE	The sum of shareholding ratios of the second to the fifth-largest shareholder/the shareholding ratio of the first-largest shareholder	CASMAR database
INDEP	Percentage of independent directors on the board.	CASMAR database
BOARD	The number of directors in the board	CASMAR database
NAF	Natural logarithm of audit fee	CASMAR database
AO	If the firm is issued an unqualified audit opinion, 1; or 0	CASMAR database
BIG4	If the firm is audited by “Big4” auditor, 1; or 0	CASMAR database
REGION	The marketization index of the region where the office is headquartered	Wang et al., 2021

Finally, we control the specific firm (τ_i_) and the particular year (γ_t_) in these two models.

### Sample

Our sample comes from CASMAR database. To ensure the accuracy of our research, we do not consider CFOs who are also CEOs. We only consider those firms that disclose CFOs’ personal information. After that, the data are processed as follows: we (1) delete those firms whose ownership type cannot be determined, (2) delete those firms in the financial industry, (3) delete those firms that issue B shares, (4) delete those firms that are specially treated (ST), (5) select the data period from 2009 to 2017, and (6) delete those observations with missing data.

After these steps, we collect a total of 15,840 observations. To eliminate the noise caused by outliers, we winsorize all continuous variables at the 1st and 99th percentiles.

In addition, except for the data on REGION, which come from Wang et al. (2021),^1^ all other data are based on or directly from the CASMAR database.

### Data Description

Table 2 presents the data description. We show them separately by ownership type. As shown in Table 2, there are large differences between SOEs and PEs. On average, it is timelier for SOEs to disclose their annual reports. A comparison of demographic characteristics shows that CFOs at SOEs have more diverse functional experiences (DIV), with a larger average age (AGE) or average tenure (TEN). However, the proportion of directors (DIR) is relatively small for CFOs at SOEs, and there is a smaller percentage of CPAs for them.

**TABLE 2:** Descriptive statistics.

	SOEs (*N* = 6,522)				PEs (*N* = 9,318)				
	P10	P50	P90	SD	P10	P50	P90	SD	Mean diff
DELAY1	70	90	117	18.7	69	101	117	20.3	–3.570***
DELAY2	69	90	117	18.8	69	101	117	20.1	–3.554***
DIV	0	1	2	0.761	0	1	2	0.753	0.043***
TEN	1	4	10	3.47	1	3	7	2.71	0.933***
AGE	39	46	54	5.91	37	44	53	6.31	2.227***
DIR	0	0	1	0.402	0	0	1	0.445	–0.070***
CPA	0	0	0	0.281	0	0	1	0.387	–0.097***
SIZE	21	22.5	24.5	1.34	20.5	21.6	23.1	1.04	0.901***
LEVER	0.241	0.526	0.769	0.197	0.126	0.371	0.66	0.2	0.132***
ROA	–0.00204	0.0283	0.0884	0.0483	0.004	0.0415	0.103	0.0497	–0.013***
INTAN	0.00252	0.0335	0.115	0.0618	0.00533	0.0344	0.0894	0.0467	0.006***
INVEN	0.0128	0.121	0.373	0.161	0.0263	0.117	0.307	0.142	0.011***
REC	0.00414	0.0544	0.214	0.0937	0.0106	0.111	0.269	0.102	–0.042***
BADNEWS	0	1	1	0.498	0	1	1	0.496	–0.012
LOSS	0	0	1	0.306	0	0	0	0.26	0.032***
SUBS	1.1	2.48	3.69	1.01	1.1	2.2	3.5	0.997	0.199***
MKBK	1.15	2.48	6.1	2.86	1.67	3.46	8.08	3.37	–1.123***
LARGEST	19.9	38.8	60.1	15.3	16.3	30.8	52.4	14.3	6.271***
BALANCE	0.058	0.288	1.13	0.484	0.147	0.623	1.65	0.606	–0.302***
INDEP	33.3	33.3	42.9	5.31	33.3	33.3	42.9	5.2	–0.659***
BOARD	7	9	11	1.9	7	9	9	1.49	1.021***
NAF	12.9	13.7	14.9	0.825	12.9	13.5	14.3	0.564	0.329***
AO	1	1	1	0.146	1	1	1	0.16	0.004*
BIG4	0	0	0	0.295	0	0	0	0.156	0.071***
REGION	4.94	7.44	9.73	1.8	6.03	8.89	9.97	1.61	–0.858***

*, **, and *** denote statistical significance at the 10%, 5%, and 1% levels (two-tailed).

## Empirical Results

### Basic Test Results

Tables 3, 4 show the test results for SOEs. Table 3 uses DELAY1 as the dependent variable. Table 4 uses DELAY2 as the dependent variable. In both tables, the explanatory variable DIV shows positive statistical significance. This means that the more diverse the functional experience of CFOs at SOEs, the less timely the disclosure of annual reports. Hypothesis 1 is thus confirmed.

**TABLE 3:** CFO functional diversity and timeliness for SOEs (DELAY1).

	(1)		(2)		(3)		(4)	
DIV	1.246***	(2.66)	1.104**	(2.45)	1.695***	(2.99)	1.145**	(2.52)
TEN			–0.024	(–0.24)	–0.019	(–0.19)	–0.022	(–0.21)
AGE			0.045	(0.64)	0.056	(0.75)	0.039	(0.55)
DIR			–0.675	(–0.69)	–0.734	(–0.75)	–0.573	(–0.59)
CPA			–1.133	(–0.95)	–1.142	(–0.95)	–1.124	(–0.94)
SIZE			3.784***	(3.57)	3.741***	(3.52)	3.705***	(3.51)
LEVER			–4.779	(–1.24)	–4.799	(–1.25)	–4.464	(–1.16)
ROA			–24.106**	(–2.49)	–24.434**	(–2.53)	–23.622**	(–2.43)
INTAN			12.441	(1.34)	12.719	(1.38)	13.130	(1.45)
INVEN			2.558	(0.46)	2.201	(0.39)	2.249	(0.40)
REC			6.987	(0.79)	6.550	(0.73)	6.790	(0.76)
BADNEWS			1.052**	(2.43)	1.017**	(2.35)	1.059**	(2.46)
LOSS			2.816***	(2.86)	2.832***	(2.87)	2.853***	(2.90)
SUBS			1.290*	(1.73)	1.290*	(1.72)	1.318*	(1.77)
MKBK			–0.238	(–1.46)	–0.243	(–1.49)	–0.238	(–1.48)
LARGEST			0.091	(1.45)	0.088	(1.40)	0.096	(1.53)
BALANCE			3.156**	(2.55)	3.127**	(2.53)	3.209***	(2.60)
INDEP			0.124	(1.48)	0.124	(1.49)	0.125	(1.49)
BOARD			0.735**	(2.51)	0.723**	(2.46)	0.729**	(2.49)
NAF			2.478**	(2.44)	2.440**	(2.42)	2.438**	(2.41)
AO			–8.254***	(–3.46)	–8.222***	(–3.45)	–8.122***	(–3.43)
BIG4			–0.049	(–0.02)	–0.125	(–0.06)	0.031	(0.01)
REGION			–0.632	(–1.03)	–0.616	(–1.01)	–0.649	(–1.06)
INCEN1					1.982*	(1.83)		
INCEN1 * DIV					–1.363*	(–1.76)		
INCEN2							8.228***	(2.90)
INCEN2* DIV							–3.517**	(–2.13)
Constant	86.842***	(98.03)	–36.557*	(–1.67)	–36.116*	(–1.65)	–34.687	(–1.59)
Year FE	Yes		Yes		Yes		Yes	
Firm FE	Yes		Yes		Yes		Yes	
Observations	6,522		6,522		6,522		6,522	
Adjusted R-squared	0.042		0.077		0.078		0.078	

*, **, and *** denote statistical significance at the 10, 5, and 1% levels (two-tailed), respectively. The standard errors are clustered by firm, and the t-statistics are reported in parentheses.

**TABLE 4:** CFO functional diversity and timeliness for SOEs (DELAY2).

	(1)		(2)		(3)		(4)	
DIV	1.383***	(3.15)	1.270***	(2.99)	2.009***	(3.89)	1.317***	(3.10)
TEN			–0.014	(–0.14)	–0.011	(–0.11)	–0.013	(–0.13)
AGE			0.028	(0.38)	0.048	(0.62)	0.023	(0.32)
DIR			–0.750	(–0.79)	–0.822	(–0.87)	–0.655	(–0.69)
CPA			–0.994	(–0.83)	–1.040	(–0.87)	–0.994	(–0.83)
SIZE			2.854***	(2.81)	2.800***	(2.75)	2.772***	(2.73)
LEVER			–4.802	(–1.27)	–4.844	(–1.28)	–4.486	(–1.19)
ROA			–22.568**	(–2.30)	–23.070**	(–2.36)	–22.131**	(–2.25)
INTAN			10.650	(1.10)	11.003	(1.15)	11.316	(1.21)
INVEN			3.789	(0.71)	3.299	(0.62)	3.538	(0.66)
REC			7.251	(0.82)	6.670	(0.75)	7.087	(0.80)
BADNEWS			0.710	(1.62)	0.668	(1.53)	0.719	(1.64)
LOSS			3.049***	(3.20)	3.069***	(3.22)	3.082***	(3.24)
SUBS			0.989	(1.32)	0.984	(1.31)	1.016	(1.36)
MKBK			–0.354**	(–2.06)	–0.361**	(–2.09)	–0.354**	(–2.08)
LARGEST			0.022	(0.37)	0.017	(0.30)	0.027	(0.46)
BALANCE			1.742	(1.50)	1.699	(1.47)	1.795	(1.55)
INDEP			0.078	(0.95)	0.078	(0.95)	0.078	(0.96)
BOARD			0.730**	(2.29)	0.718**	(2.25)	0.724**	(2.27)
NAF			3.480***	(3.47)	3.438***	(3.45)	3.449***	(3.44)
AO			–4.968*	(–1.94)	–4.923*	(–1.91)	–4.831*	(–1.88)
BIG4			0.460	(0.19)	0.386	(0.16)	0.524	(0.22)
REGION			–1.104*	(–1.78)	–1.082*	(–1.75)	–1.120*	(–1.81)
INCEN1					2.739***	(2.67)		
INCEN1* DIV					–1.705**	(–2.31)		
INCEN2							8.016***	(2.66)
INCEN2* DIV							–3.685**	(–2.09)
Constant	86.838***	(98.63)	–22.728	(–1.06)	–22.623	(–1.05)	–20.960	(–0.98)
Year FE	Yes		Yes		Yes		Yes	
Firm FE	Yes		Yes		Yes		Yes	
Observations	6,522		6,522		6,522		6,522	
Adjusted R-squared	0.038		0.066		0.068		0.067	

*, **, and *** denote statistical significance at the 10, 5, and 1% levels (two-tailed), respectively. The standard errors are clustered by firm, and the t-statistics are reported in parentheses.

It can be seen from INCEN1*DIV or INCEN2*DIV that promotion incentive can play a negative moderating role. Hypothesis 2 is thus confirmed. In addition, the incentive effects produced by political connections (INCEN1) seems greater than those produced by human capital advantage (INCEN2) if other variables remain unchanged.

The marketization index is usually between 0 and 10. We define regions with a marketization index of less than eight as less developed regions and all others as developed regions. This classification is based on the fact that most public firms, especially listed PEs, are located in developed regions.^2^

Tables 5, 6 show the test results for PEs. It can be seen from Models (1) and (2) that the coefficient of DIV becomes negative. Models (3) and (4) are test results for different regions. In Model (3), the DIV becomes larger and statistically significant. This means that in regions with a low level of marketization, the more diverse the functional experience of CFOs at PEs, the timelier the disclosure of annual reports. Hypothesis 3 is thus confirmed.

**TABLE 5:** CFO functional diversity and timeliness for PEs (DELAY1).

	Total				Less developed		Others	
	(1)		(2)		(3)		(4)	
DIV	–0.696	(–1.43)	–0.596	(–1.27)	–2.207***	(–2.71)	–0.184	(–0.32)
TEN			–0.072	(–0.55)	0.051	(0.22)	–0.255	(–1.49)
AGE			–0.012	(–0.17)	–0.073	(–0.59)	–0.031	(–0.33)
DIR			–0.245	(–0.31)	0.095	(0.07)	0.473	(0.48)
CPA			–1.994**	(–2.15)	–1.384	(–0.81)	–2.760**	(–2.37)
SIZE			1.176	(1.25)	4.511***	(2.61)	0.399	(0.34)
LEVER			0.220	(0.08)	0.024	(0.00)	–0.284	(–0.08)
ROA			–36.007***	(–4.04)	–54.649***	(–3.41)	–19.492*	(–1.70)
INTAN			2.751	(0.34)	–13.757	(–0.97)	12.545	(1.38)
INVEN			–1.873	(–0.49)	–12.316	(–1.53)	2.214	(0.45)
REC			13.261**	(2.46)	–0.141	(–0.01)	15.159**	(2.09)
BADNEWS			2.060***	(4.75)	2.662***	(3.30)	2.385***	(4.51)
LOSS			3.266***	(2.86)	3.320*	(1.82)	3.229**	(2.04)
SUBS			0.909	(1.44)	1.418	(1.21)	0.425	(0.54)
MKBK			–0.743***	(–5.94)	–0.684***	(–2.89)	–0.561***	(–3.70)
LARGEST			–0.173***	(–3.06)	–0.058	(–0.58)	–0.196**	(–2.45)
BALANCE			–2.191**	(–1.98)	–1.005	(–0.48)	–1.971	(–1.44)
INDEP			0.055	(0.62)	0.155	(0.91)	–0.010	(–0.10)
BOARD			0.252	(0.68)	–0.249	(–0.34)	0.134	(0.29)
NAF			2.523**	(2.26)	–1.152	(–0.58)	4.176***	(3.12)
AO			–12.144***	(–7.80)	–12.398***	(–4.59)	–10.080***	(–5.39)
BIG4			2.909	(1.01)	11.399***	(4.30)	3.256	(1.12)
REGION			–0.975*	(–1.92)	–1.303	(–0.81)	0.096	(0.12)
Constant	85.357***	(66.81)	53.318***	(2.62)	33.266	(0.98)	40.665	(1.62)
Year FE	Yes		Yes		Yes		Yes	
Firm FE	Yes		Yes		Yes		Yes	
Observations	9,318		9,318		3,441		5,877	
Adjusted R-squared	0.056		0.101		0.101		0.091	

*, **, and *** denote statistical significance at the 10, 5, and 1% levels (two-tailed). The standard errors are clustered by firm, and the t-statistics are reported in parentheses. “LESS DEVELOPED” indicates firms whose offices are headquartered in regions where the total marketization index is less than 8.

**TABLE 6:** CFO functional diversity and timeliness for PEs (DELAY2).

	Total				Less developed		Others	
	(1)		(2)		(3)		(4)	
DIV	–0.327	(–0.67)	–0.215	(–0.45)	–1.735**	(–2.06)	0.151	(0.27)
TEN			–0.122	(–0.90)	–0.015	(–0.07)	–0.276	(–1.60)
AGE			–0.075	(–1.10)	–0.131	(–1.05)	–0.087	(–0.99)
DIR			0.268	(0.34)	0.757	(0.55)	0.914	(0.91)
CPA			–1.349	(–1.43)	–2.008	(–1.15)	–1.510	(–1.30)
SIZE			1.242	(1.35)	4.316***	(2.67)	0.172	(0.15)
LEVER			–0.586	(–0.20)	0.503	(0.09)	–0.740	(–0.22)
ROA			–41.508***	(–4.72)	–51.239***	(–3.37)	–30.414***	(–2.68)
INTAN			7.719	(1.01)	–9.558	(–0.73)	16.654*	(1.87)
INVEN			–2.220	(–0.61)	–14.246**	(–1.97)	1.879	(0.40)
REC			17.497***	(3.12)	2.733	(0.28)	19.817***	(2.65)
BADNEWS			1.433***	(3.40)	1.809**	(2.29)	1.729***	(3.37)
LOSS			1.662	(1.53)	1.572	(0.98)	1.770	(1.13)
SUBS			1.265**	(2.02)	1.120	(1.00)	1.226	(1.54)
MKBK			–0.663***	(–5.27)	–0.797***	(–3.33)	–0.384***	(–2.61)
LARGEST			–0.181***	(–3.35)	–0.065	(–0.72)	–0.174**	(–2.27)
BALANCE			–2.396**	(–2.24)	–1.431	(–0.74)	–2.139	(–1.61)
INDEP			0.080	(0.93)	0.318**	(2.01)	–0.042	(–0.41)
BOARD			0.204	(0.55)	0.255	(0.36)	–0.281	(–0.60)
NAF			1.015	(0.89)	–2.720	(–1.35)	2.437*	(1.83)
AO			–7.231***	(–4.66)	–8.576***	(–3.31)	–5.316**	(–2.57)
BIG4			2.425	(0.81)	7.725***	(2.95)	1.763	(0.51)
REGION			–1.189**	(–2.42)	–1.812	(–1.16)	–0.257	(–0.35)
Constant	85.690***	(68.69)	70.317***	(3.57)	50.910	(1.54)	71.452***	(2.96)
Year FE	Yes		Yes		Yes		Yes	
Firm FE	Yes		Yes		Yes		Yes	
Observations	9,318		9,318		3,441		5,877	
Adjusted R-squared	0.047		0.081		0.079		0.073	

*, **, and *** denote statistical significance at the 10, 5, and 1% levels (two-tailed). The standard errors are clustered by firm, and the t-statistics are reported in parentheses. “LESS DEVELOPED” indicates firms whose offices are headquartered in regions where the total marketization index is less than 8.

Overall, the relationships between functional diversify and the timeliness of annual reports are different under different institutional backgrounds. It should be noted that if the research subjects in Tables 3–6 are exchanged, the conclusions will be completely different. On the one hand, the moderating effect associated with promotion incentive will no longer be statistically significant for PEs. On the other hand, in regions where the level of marketization is high (rather than low), the more diverse the functional experience of CFOs at SOEs, the less (rather than more) timely the disclosure of annual reports. This is consistent with promotion logic. In regions with a high level of marketization, the promotion opportunity for generalist CFOs at SOEs is relatively low. As a result, they may show significant inefficiency.

### Sensitive Analysis

We use the difference-in-differences (DID) method to alleviate the endogenous problem. We select those CFOs who have experienced functional diversification as the treatment group (treat = 1) and those who have not as the control group (treat = 0). For the treatment group, we set POST^3^ to 0 before CFOs experienced functional diversification and to 1 or above afterward, depending on the number of functional areas added (i.e., POST = DIV).


(3) $${\text{DELAY}}_{\text{i},\text{t}}=\beta_{0}+\beta_{1}\,{\text{TRAET}}_{\text{i},\text{t}}*{\text{POST}}_{\text{i},\text{t}}+\Sigma \,\rho_{\text{i}}\,{\text{CONTROL}}_{\text{i},\text{t}}+\tau_{\text{i}}+\gamma_{\text{t}}+\omega_{\text{i},\text{t}}$$

Model (3) is the DID model. If β_**1**_ is positive, it means that the CFO’s functional diversification is positively associated with a delay in annual reports. Otherwise, the relationship becomes negative.

Table 7 shows the test results, which are similar to the above tables. Thus, the above hypotheses are confirmed again.

**TABLE 7:** DID tests for CFO functional diversification and timeliness (DELAY1).

	SOEs				PEs			

					Less developed		Others	
	(1)		(2)		(3)		(4)	
POST*TREAT	2.247**	(2.18)	1.830*	(1.83)	–2.895*	(–1.67)	–1.267	(–0.93)
TEN			–0.086	(–0.34)	–0.002	(–0.00)	–0.724	(–1.53)
AGE			0.139	(0.59)	–0.338	(–1.24)	–0.038	(–0.15)
DIR			–1.862	(–0.92)	0.303	(0.12)	1.556	(0.76)
CPA			–2.485	(–1.24)	0.129	(0.04)	–3.823	(–1.49)
SIZE			6.526***	(3.71)	3.585	(1.24)	1.235	(0.60)
LEVER			–2.886	(–0.48)	5.738	(0.62)	0.177	(0.03)
ROA			–34.658*	(–1.85)	–50.184*	(–1.93)	–38.626*	(–1.77)
INTAN			–4.371	(–0.30)	10.996	(0.43)	9.705	(0.65)
INVEN			2.304	(0.27)	–19.763	(–1.55)	–4.148	(–0.51)
REC			12.301	(1.11)	–8.414	(–0.44)	13.468	(1.05)
BADNEWS			1.174	(1.53)	1.994	(1.47)	3.032***	(3.47)
LOSS			4.143**	(2.53)	3.132	(1.14)	2.997	(1.17)
SUBS			–0.150	(–0.13)	2.715	(1.41)	1.474	(1.07)
MKBK			–0.486	(–1.50)	–0.509	(–1.11)	–0.506*	(–1.92)
LARGEST			0.214*	(1.89)	0.063	(0.44)	–0.128	(–1.07)
BALANCE			2.636	(1.41)	5.417**	(2.07)	0.222	(0.11)
INDEP			0.014	(0.10)	0.431*	(1.67)	–0.167	(–0.95)
BOARD			0.927*	(1.77)	0.664	(0.57)	–0.242	(–0.26)
NAF			0.955	(0.44)	–4.026	(–1.20)	1.704	(0.77)
AO			–2.902	(–0.56)	–11.236***	(–3.01)	–14.240***	(–3.96)
BIG4			–3.282	(–0.83)	16.128***	(5.14)	–4.755	(–1.15)
REGION			–1.925*	(–1.74)	–1.485	(–0.55)	1.515	(1.09)
Constant	86.814***	(64.15)	–74.477*	(–1.86)	64.831	(1.12)	55.085	(1.19)
Year FE	Yes		Yes		Yes		Yes	
Firm FE	Yes		Yes		Yes		Yes	
Observations	2,194		2,194		1,428		2,287	
Adjusted R-squared	0.041		0.086		0.095		0.091	

*, **, and *** denote statistical significance at the 10, 5, and 1% levels (two-tailed). The standard errors are clustered by firm, and the t-statistics are reported in parentheses. “LESS DEVELOPED” indicates firms whose offices are headquartered in regions where the marketization index is less than 8.

We may ignore some variables that affect both the explanatory and dependent variables. We thus add LAG_DELAY (the lagged term of DELAY1), SALARY (the natural logarithm of the CFO’s salary), BUSY (the number of directors served by the CFO at other firms), and ADVANCE (whether there is an issuance of financial forecasts related to the current annual report). After adding these variables, the conclusions remain robust.

## Summary and Comments

Through research on Chinese listed firms, we found that, in SOEs, the more diverse the functional experience, the less timely the disclosure of annual reports. This negative link can be weakened if generalist CFOs have more chance of promotion. In PEs, the more diverse the functional experience, the timelier the disclosure of annual reports. It should be noted that this relationship only occurs for firms in less developed regions, where generalist CFOs can fully use their complementary knowledge or wide social networks to prepare financial statements.

Our findings suggest a large difference in institutional background between SOEs and PEs (Conyon and He, 2014), and this difference can profoundly affect the behavioral choice made by those generalist CFOs. To some extent, functional diversification can help CFOs improve their work efficiency. In PEs, we found such evidence. However, in SOEs, the test results gave the opposite answer. We believe these results are closely related to SOEs’ personnel policies. There is no efficient employee exit mechanism in SOEs. As a result, CFOs with broad social networks do not worry about career security. Moreover, if they have less chance of promotion, they will rely on their social capital to slack off.

Our findings also imply that in SOEs, CFOs’ functionally diverse experience leads to their lack of identity as heads of accounting departments and makes the CFOs more like political officials rather than professional managers (Hu and Xu, 2021). Many scholars have found that, in China, CEOs with different labels have different impacts on firms’ financial policies (Fan et al., 2007; Li and Qian, 2013), especially for those at SOEs (Xie, 2015; Fang et al., 2018; Lou et al., 2021). This paper thus expands the research scope and provides an understanding of other executives in Chinese SOEs.

Our evidence on PEs shows that generalist executives can only demonstrate their complementary value as human capital under certain circumstances. This is consistent with the views of other scholars (Li and Patel, 2019; Mueller et al., 2021). In addition, few scholars have explored the link between institutions and executive behavior (Krause et al., 2019). Through comparative study, this paper increases the relevant knowledge.

Most scholars use upper echelons theory to link executive characteristics with corporate strategy or firm performance. However, this theory is premised on the assumption that institutions allow executives to be willing and able to demonstrate their personalities or values. Otherwise, the intrinsic preferences of executives will be hidden, and the relationship between personal characteristics and explained variables cannot be reasonably established. Our empirical results show that it seems more reasonable to use agency theory to explain the behavior of SOE executives. Our findings highlight the constraints imposed by institutions, which may be worth considering for future research.

## Limitations

We tried to determine the reasons behind the positive or negative relationship between functional diversity and the timeliness of annual reports. We found that promotion incentive or the level of marketization can play a role. There may be other factors that influence the attitudes of generalist CFOs toward financial reporting, such as the monitoring role of public media, the pressure from block holders, and so on.

The annual report involves many sections, and it is not just the CFO who needs to be involved in its preparation. The CEO or the board of directors may have more say in the timing of disclosure. Therefore, there are many issues to be further explored.

We also did not completely address the endogeneity problem. We cannot find suitable instrumental variables that represent the explanatory variable and are linked with different ownership types. Thus, this constitutes a shortcoming of our study.

## Data Availability Statement

The original contributions presented in the study are included in the article/supplementary material, further inquiries can be directed to the corresponding author/s.

## Author Contributions

HY was a supervisor for the writing and improvement of the manuscript. WH was responsible for the idea, data collection and hypothesis test. Both authors contributed to the article and approved the submitted version.

## Conflict of Interest

The authors declare that the research was conducted in the absence of any commercial or financial relationships that could be construed as a potential conflict of interest.

## Publisher’s Note

All claims expressed in this article are solely those of the authors and do not necessarily represent those of their affiliated organizations, or those of the publisher, the editors and the reviewers. Any product that may be evaluated in this article, or claim that may be made by its manufacturer, is not guaranteed or endorsed by the publisher.

## Footnotes

1 In Wang’s book, the marketization data for 2008–2016 and 2016–2019 are not directly comparable. Based on the marketization data of 2008–2016, we adjust the data of 2017 to make all data comparable.

2 For the robustness of our conclusions, we reclassify the regions and define regions with a marketization index of less than 7 or 6 as less developed regions. The test results also support our hypothesis.

3 There is a technical problem. To include the control group into the sample, we need set POST to 0 for the control group.
